# A Framework for Predicting Winter Wheat Yield in Northern China with Triple Cross-Attention and Multi-Source Data Fusion

**DOI:** 10.3390/plants14142206

**Published:** 2025-07-16

**Authors:** Shuyan Pan, Liqun Liu

**Affiliations:** College of Information Science and Technology, Gansu Agricultural University, Lanzhou 730070, China

**Keywords:** multi-source features, cross-attention mechanism, yield forecast, Transformer, graph attention mechanism

## Abstract

To solve the issue that existing yield prediction methods do not fully capture the interaction between multiple factors, we propose a winter wheat yield prediction framework with triple cross-attention for multi-source data fusion. This framework consists of three modules: a multi-source data processing module, a multi-source feature fusion module, and a yield prediction module. The multi-source data processing module collects satellite, climate, and soil data based on the winter wheat planting range, and constructs a multi-source feature sequence set by combining statistical data. The multi-source feature fusion module first extracts deeper-level feature information based on the characteristics of different data, and then performs multi-source feature fusion through a triple cross-attention fusion mechanism. The encoder part in the production prediction module adds a graph attention mechanism, forming a dual branch with the original multi-head self-attention mechanism to ensure the capture of global dependencies while enhancing the preservation of local feature information. The decoder section generates the final predicted output. The results show that: (1) Using 2021 and 2022 as test sets, the mean absolute error of our method is 385.99 kg/hm^2^, and the root mean squared error is 501.94 kg/hm^2^, which is lower than other methods. (2) It can be concluded that the jointing-heading stage (March to April) is the most crucial period affecting winter wheat production. (3) It is evident that our model has the ability to predict the final winter wheat yield nearly a month in advance.

## 1. Introduction

Wheat production and supply are critical to maintaining global food security. Forecasting winter wheat production has always been one of the key issues in agricultural scientific research. How to accurately and timely predict a winter wheat yield is crucial for safeguarding national food security and plays a significant role in optimizing global agricultural development strategies and tackling the challenges of climate change. Remote sensing image refers to the image data that is converted by using different sensors to receive and record electromagnetic wave information from different objects. Due to different sensors, it can be further divided into hyperspectral imaging and multispectral imaging. Hyperspectral remote sensing images typically contain a large number of spectral bands that can provide rich spectral information, but their spatial resolution is low and difficult to obtain. Multispectral remote sensing images usually have high spatial resolution and include different bands such as visible light, near-infrared, and short-wave infrared. They can be used to calculate satellite remote sensing features such as vegetation index (VI), and then monitor the growth process of winter wheat, which is relatively easy to obtain. Using only a single VI to predict a winter wheat yield cannot provide comprehensive information about its growth process. Therefore, this article adopts a multi-source feature fusion approach, focusing on satellite remote sensing features, climate features, and soil features during the growth process of winter wheat. The focus is on capturing the both intra-feature and inter-feature relationships of different features, in order to accurately predict winter wheat yields in northern China.

### 1.1. Related Work

Current methods commonly used for winter wheat yield prediction fall into three broad categories: methods based on crop simulation models, methods based on traditional machine learning, and methods based on deep learning.

(1) The method based on crop simulation models focuses on the growth process of crops combined with biological principles, using environmental data such as climate and soil, and crop data like photosynthesis and transpiration, to simulate crop growth and predict the final yield. Common models for crop modeling include the Agricultural Production Systems sIMulator (APSIM), the Decision Support System for Agrotechnology Transfer (DSSAT), and the Crop Simulation Model for Agricultural Management Decision Support (CropSyst). After collecting data related to crop growth, researchers need to select appropriate biophysical models and parameterize the models based on local conditions, then use these parameters to simulate the crop growth process through the selected model. They should then compare the predicted production data obtained from the simulation process with the actual production data and further adjust the model to improve prediction precision. Zhao et al. (2024) [1] suggested a model on the basis of APSIM for simultaneously predicting wheat and corn yields, which can analyze the relationship between wheat and corn yields and environmental factors. Zhao et al. (2022) [2] simulated various indicators such as cumulative biomass using APSIM and used them as inputs for statistical regression models to ultimately predict wheat yield. Uvirkaa Akumaga et al. (2023) [3] combined high-resolution remote sensing satellite data, observation data from the ground, and DSSAT to simulate the number of days during the partial growth stages of soybeans and corn, and ultimately the estimated yield. Yang et al. (2023) [4] proposed a simulation method for a pre-season crop yield prediction for corn, combined with DSSAT. Yang et al. (2023) [5] used DSSAT to evaluate the trend of maize yield changes with future climate change, and analyzed the changes in future yield and the reasons for these changes through experiments. Harsimran Kaur et al. (2022) [6] combined CropSyst with historical and recent climate data for yield prediction, and identified spring peas as the optimal elastic crop, which can help improve the sustainability of crop rotation systems. Simone Bregaglio et al. (2023) [7] proposed a method for predicting yield by combining time series remote sensing data and agricultural models. The above methods based on crop simulation models require high-quality input data and are costly to construct and calibrate.
(2) The method based on traditional machine learning analyzes the data of different features by combining domain knowledge to further excavate the key factors that have a greater impact on crop yield and build a model for prediction. The commonly used traditional machine learning models include Linear Regression (LR), Support Vector Regression (SVR), Random Forest (RF), Gradient Boosting Decision Tree (GBDT), and eXtreme Gradient Boosting (XGBoost). Fei et al. (2024) [8] evaluated the performance of three methods, including RF, in predicting wheat yield using hyperspectral reflectance data from early- and mid-grain filling during wheat growth. Li et al. (2023) [9] established a soybean yield prediction model by integrating K-nearest neighbors, RF, and SVR through ensemble learning. Sun et al. (2024) [10] applied partial least squares regression, RF, and SVR to assess the connection between multi- vegetation indices and the yield of three types of rice at different growth stages. Li et al. (2023) [11] proposed a soybean yield forecast framework that combines XGBoost and multidimensional feature engineering. Yu et al. (2023) [12] proposed a meta learning ensemble regression framework that combines optical data, synthetic aperture radar data, and meteorological data to accurately predict rice yield. Diego Arruda Huggins de Sá Leitão et al. (2023) [13] compared different prediction methods and found that algorithms such as RF and SVR performed better than traditional linear regression methods. Moreover, traditional regression methods had overestimation and underestimation biases when predicting low-yield and high-yield areas. Zhang et al. (2023) [14] suggested a predictive model using Bayesian optimized Categorical Boosting (CatBoost) based on Landsat-8 and Sentinel-2 vegetation index time series data to estimate a winter wheat yield. Wang et al. (2023) [15] applied RF and other methods to study the relationship between three different satellite data and maize yield under different conditions of normal and drought years. Juan Skobalaski et al. (2024) [16] used methods such as RF and Gradient Boosting Regression (GBR) to predict soybean yield and proposed a novel transfer learning approach to the selection of genotypes and high-yield variety screening. Cheng et al. (2022) [17] assessed the effectiveness of RF, GBDT, SVR, and deep learning methods for predicting yield using multispectral, hyperspectral, and gridded yield data. The model construction and training process of the methods based on traditional machine learning mentioned above is relatively simple, but it usually requires manual feature selection before training the model, and the quality of the input data is critical to model performance.
(3) The method based on deep learning is adept at capturing nonlinear relationships, complex patterns, and long-term dependencies between features through neural network models. By automatically extracting features, different weights are assigned to different features, ultimately achieving yield prediction. The commonly used models include Convolutional Neural Network (CNN), Long Short Term Memory (LSTM), Gated Recurrent Unit (GRU), Recurrent Neural Network (RNN), and Transformer. Wang et al. (2023) [18] proposed a model named CNN-GRU using three different remote sensing variables to estimate a winter wheat yield. Guo et al. (2023) [19] collected multispectral and hyperspectral images from ground measurements as model inputs, and used CNN and other models to predict a corn yield separately. Feng et al. (2024) [20] used CNN to extract soil features, meteorological features, and image features captured by drones, and then used GRU for yield prediction. Mahdiyeh Fathi et al. (2023) [21] proposed a model called 3D-ResNet-BiLSTM for predicting soybean yields. Tanabe et al. (2023) [22] used hyperspectral technology and CNN to assess the impact of four growing stages of winter wheat on predicting yield. Bi et al. (2023) [23] introduced a yield prediction model utilizing Transformer to comprehensively incorporate image features and seed features. Gregor Perich et al. (2023) [24] proposed a method for precise agricultural modeling using Sentinel-2 satellite data. The study selected and analyzed three mainstream methods: data analysis methods based on spectral indices, raw satellite reflectance, and RNN. The results indicated that the performance of RNN may not necessarily be superior to other methods, but it is more effective due to its end-to-end training approach. Cheng et al. (2024) [25] proposed a county-level winter wheat yield prediction method called GT-LSTM to address the difficulty of learning geographic spatial information in using RNN to process crop time-series data. Guo et al. (2024) [26] proposed a model called SSA-LSTM-transformer using multiple remote sensing variables to predict wheat yield. The model combines the automatic optimization capability of sparrow search algorithm with the long-term memory ability of LSTM. Kiran Kumar et al. (2023) [27] optimized hyperparameter configuration and fine-tuned LSTM and bidirectional LSTM to achieve the yield prediction of crops such as wheat. The above methods based on deep learning can automatically extract important features, reduce manual intervention, and are suitable for processing complex high-dimensional data, but the methods usually require a large amount of computation.

### 1.2. Existing Problems and Advantages

The existing winter wheat yield prediction methods have the following problems. Firstly, they generally rely heavily on domain knowledge, have a poor generalization ability of model assumptions, and require manual feature selection. Secondly, the interaction between multiple factors has not been fully utilized, and most methods rely on statistical models, resulting in low prediction accuracy and poor adaptability.

To address these limitations, this paper proposes a winter wheat yield prediction framework with triple cross-attention and multi-source data fusion to fully utilize multi-source feature information for the accurate prediction of winter wheat yields. Compared with existing methods, our approach has the following advantages. To begin with, an accurate yield prediction for large-scale crop planting areas such as districts and counties can provide a basis for government decision-making. In addition, using multi-source data to predict a yield can fully account for the impact of different factors as the crop grows. Moreover, using deep learning methods to achieve a yield prediction can adaptively assess the influence of various features on the yield, without the need for traditional feature screening, thereby reducing the negative effects of manual intervention. Lastly, the difference between our model and other Transformerbased models is that our model introduces a graph attention mechanism to enhance the capture of local features, and will extract temporal information from different features in advance and adaptively fuse them.

### 1.3. Contributions

The key contributions of this paper are summarized as follows:

(1) We suggest a winter wheat yield prediction framework with triple cross-attention and multi-source data fusion. This framework consists of three modules, namely a multi-source data processing module, a multi-source feature fusion module, and a yield prediction module.
(2) The multi-source data processing module obtains raw data through different platforms and combines relevant software to extract multi-source data, ultimately generating a sequence set corresponding to the multi-source data.
(3) Different approaches are used to capture the internal information of data from different sources. For dynamic features that change over time during the growing period, a Temporal Feature Enhancement Module (TFE) is proposed to capture the temporal information. For static soil features that are almost unchanged during the growing period, a Convolutional Residual Block (CRB) is used to extract the deep features.
(4) In order to fuse the extracted multi-source features, this paper proposes a novel fusion method called Triple Cross-Attention Fusion Mechanism (TCAFM), which captures the relationship between multi-source features while realizing multi-source feature fusion.
(5) In the yield prediction module, the encoder uses the multi-head self-attention mechanism (MHSA) and the graph attention mechanism to construct a double branch, which allows the model to capture global dependencies in the features and enhances the transfer of local information. The decoder employs Fourier Analysis Networks (FAN) to capture the complex nonlinear interactions among the processed features and the predicted yield.

### 1.4. The Structure of This Paper

The structure of this paper is as follows: The first part is the introduction, which summarizes the recent research status, existing problems, advantages of this article, and contributions of this article. The second part is materials and methods, which introduces the research area of this paper, the data used, our model and its corresponding pseudocode, and the prediction flowchart. The third part presents the results of different experiments. The fourth part is a discussion, analyzing and summarizing the results of the third part. The fifth part is the conclusion, which summarizes the content of this paper and analyzes the problems and future work of the method proposed in this paper.

## 2. Materials and Methods

### 2.1. Materials

#### 2.1.1. Research Area

This paper selects the key winter wheat production regions in Shandong Province, Shanxi Province, Henan Province, and Gansu Province (Longnan City, Tianshui City, Pingliang City, and Qingyang City) in China as the research areas.

#### 2.1.2. Data

The data used in this paper includes ground statistical data, a satellite remote sensing index, climate features, soil features, and spatio-temporal data. Detailed descriptions can be found in Table 1.

The ground statistical data used in this paper, including yield per unit area and sowing area, are all from the agricultural statistical yearbooks of each province [28]. After removing incomplete data, unit area yield data (kg·hm^−2^) and sowing area data (hm^2^) were collected for 16 cities in Shandong Province, 50 counties (districts) in Shanxi Province, 100 counties (districts) in Henan Province, and 30 counties (districts) in Gansu Province from 2001 to 2022.

A Normalized Difference Vegetation Index (NDVI) and Enhanced Vegetation Index (EVI) are frequently employed indicators extracted from satellite remote sensing data to measure the condition and extent of vegetation. This paper obtains the NDVI and EVI of the research region from the MODIS Synthetic Earth Vegetation Index product (MOD13Q1) with 250 m spatial resolution and 16 days temporal resolution [29]. The closer the NDVI value is to 1, the higher the vegetation coverage and the more vigorous the vegetation growth. As the EVI value increases, the vegetation coverage also rises, which effectively reflects crop growth. Therefore, this paper selects NDVI and EVI as the input features of the yield prediction model.

Winter wheat growth and development is significantly influenced by climate. For example, the temperature determines the time of germination, seedling stage, flowering, and the maturity of winter wheat, while precipitation affects the development and growth of winter wheat roots. This paper uses the Google Earth Engine (GEE) platform to obtain a reanalysis raster dataset called FLDAS provided by NASA [30]. The information of seven climate data is shown in Table 1.

Soil conditions are a key factor influencing crop yield, and the soil data used in this paper is divided into static soil data and dynamic soil data. Static soil data were sourced from the National Cryosphere Desert Data Center [31] in different areas studied. At the same time, the moisture and temperature of soil at different depths (0–10 cm and 10–40 cm) were extracted from the FLDAS reanalysis raster dataset as dynamic soil data. There are a total of 20 soil physicochemical properties used as soil property inputs for the prediction model, as shown in Table 1 for specific information.

### 2.2. A Winter Wheat Yield Prediction Framework with Triple Cross-Attention and Multi-Source Data Fusion

**Figure 1:**
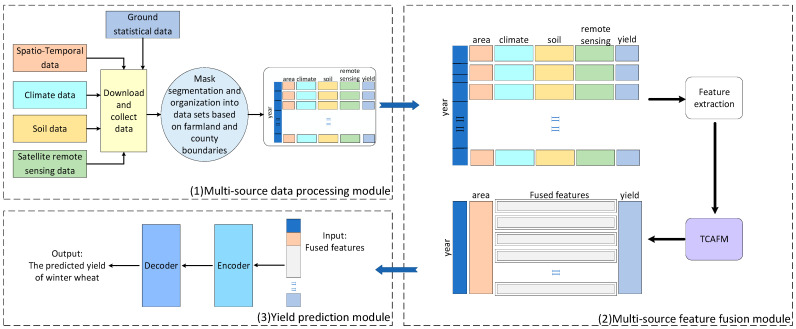
Model framework.

This paper proposes a winter wheat yield prediction framework based on triple cross-attention for multi-source data fusion, which fully utilizes multi-source feature information to achieve an accurate prediction of a winter wheat yield. The framework structure is shown in Figure 1. This framework consists of three main modules: a multi-source data processing module, a multi-source feature fusion module, and a yield prediction module. The multi-source feature fusion module is further subdivided into feature extraction and TCAFM. Firstly, we obtained multi-source features of specific regions related to yield prediction tasks on demand through different platforms and software. Secondly, based on the characteristics of different data, we chose appropriate methods to extract internal information of features. Then, TCAFM was used to capture the connections between features and enable feature fusion. Finally, the merged features were input into the yield prediction module based on the Transformer structure to complete the yield prediction task.

#### 2.2.1. Multi-Source Data Processing Module

**Figure 2:**
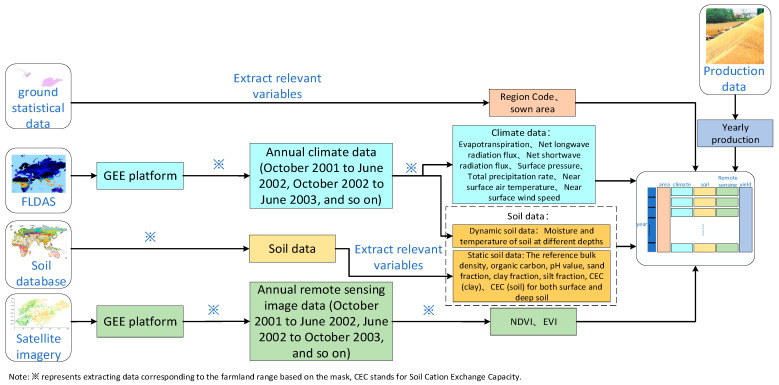
Multi-source data processing module.

Figure 2 shows the workflow of the multi-source data processing module.

This paper obtains NDVI data and EVI data corresponding to the different growing and developing stages of winter wheat in each research area through the GEE platform. Firstly, the 30 m resolution winter wheat cultivation dataset in China from 2001 to 2023, made available by the National Ecosystem Science Data Center [32], was used to identify the winter wheat planting regions within the province. Secondly, we applied quality screening using the DetailedQA band of MOD13Q1. Through bitwise operations, pixels affected by clouds, shadows, snow, and ice were masked out, effectively reducing noise and anomalies. Then, the NDVI and EVI values corresponding to the winter wheat cultivation regions were obtained from MOD13Q1 using the GEE platform, and the NDVI and EVI mean values for each study area were calculated for each month, in order to resample their time resolution to monthly units, thereby leveraging temporal averaging to mitigate the influence of sporadic noise and missing data. For a very small number of time nodes with severe missing data, we used a linear interpolation method to complete them by combining adjacent month data. Finally, the remote sensing images corresponding to the NDVI and EVI values of each month were extracted by batch mask using ArcGIS(10.8) software to obtain the NDVI data and EVI data of winter wheat planting regions within different cities or districts (counties).

The method for processing climate data was similar to that used for satellite remote sensing data. After obtaining the winter wheat planting areas within the province, the GEE platform was first used to extract the reanalysis raster data of the winter wheat planting regions from the FLDAS dataset. Then, batch masking extraction was performed using ArcGIS, and finally, the monthly average values of various climate data in the winter wheat planting areas of different cities and districts (counties) were exported.

The dynamic soil data portion of the soil data was processed using the same method as the climate data, while the static soil data portion was extracted directly from the soil dataset by mask for the specified district and county boundaries using ArcGIS.

#### 2.2.2. Multi-Source Feature Fusion Module

A multi-source feature fusion module was designed for fully leveraging the features from different sensors, combining satellite remote sensing ($x^{rs}$), climate ($x^{cl}$), and soil features (static soil features $x^{so-s}$ and dynamic soil features $x^{so-d}$). Firstly, this module performed deep level feature extraction based on the nature of the different types of features. Then, the processed features were sent to TCAFM for multi-source feature fusion.

**Figure 3:**
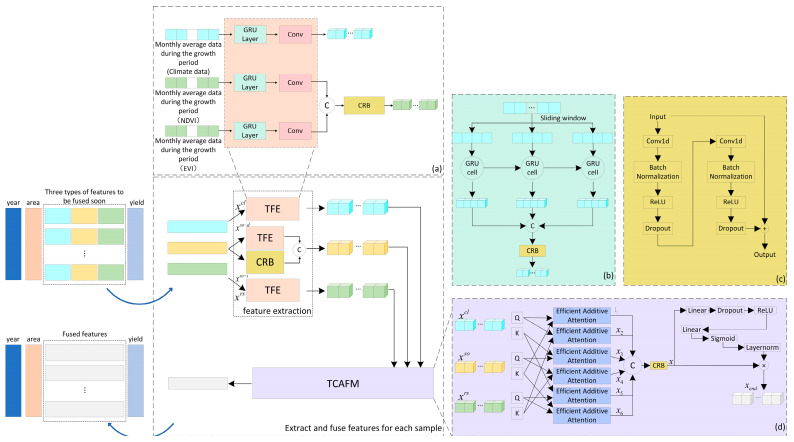
Multi-source feature fusion module. (**a**) feature extraction. (**b**) GRU Layer(taking climate data as an example). (**c**) CRB. (**d**) TCAFM.

Considering the satellite remote sensing features ($x^{rs}$), climate features ($x^{cl}$), and temperature and humidity features ($x^{so-d}$) of soil at different depths during the winter wheat growth period, which vary with time, this paper designs a TFE for capturing the internal temporal relationship of features, according to Figure 3a. The module consists of multiple GRUs connected in series and one CRB. After dividing the input features into multiple small sequences through sliding windows, the temporal features were first captured through multiple concatenated GRUs, and then deep features were extracted through a CRB. This module can enhance the internal temporal features of the features, assist the model in more accurately recognizing the periodic changes in crop growth, and improve feature accuracy and reliability.

Considering the small changes in soil physicochemical properties during the winter wheat growing season, this paper uses CRB to treat static soil features ($x^{so-s}$) to capture the complex nonlinear relationships among different soil features. CRB can automatically identify potential patterns and feature interactions, effectively extract important soil features, provide a deep understanding of soil features, and lay the foundation for subsequent analysis or application. It can then concatenate $x^{so-s}$ and $x^{so-d}$ to obtain $x^{so}$.

After processing the satellite remote sensing features, climate features, and soil features, they were sent to TCAFM for fusion. Figure 3d displays the structure of TCAFM. This fusion mechanism uses cross-attention mechanisms for satellite remote sensing features and climate features, satellite remote sensing features and soil features, and climate features and soil features. Firstly, the Query and Key corresponding to each multi-source feature are obtained through linear mapping, and then the Efficient Additive Attention mechanism [33] is used to implement a triple cross-attention mechanism, ultimately obtaining six sets of features that capture the interactions between features, as shown in Equations (1) to (3).

(1) $$Q_{i}=Linear(x^{i}),\;i=\left\{rs,\;cl,\;so\right\}$$

(2) $$K_{i}=Linear(x^{i}),\;i=\left\{rs,\;cl,\;so\right\}$$

(3) $$x_{1},x_{2},x_{3},x_{4},x_{5},x_{6}=Efficient\;Additive\;Attention(Q_{i},\;K_{i})$$

We used concatenation to concatenate 6 sets of features by channel, and then capture deep features through operations such as CRB for subsequent prediction, as shown in Equations (4) and (5).

(4) $$x=CRB(Concat(x_{1},x_{2},x_{3},x_{4},x_{5},x_{6}))$$

(5) $$x_{end}=x\times Layernorm(Sigmoid(Linear(ReLU(Dropout(Linear(x))))))$$

The use of the cross-attention mechanism for satellite remote sensing features and climate features can effectively assess how climate change influences crop health and aid in understanding the correlation between climate conditions and satellite remote sensing data. The use of the cross-attention mechanism for satellite remote sensing features and soil features can help capture how soil physicochemical properties affect crop growth. The use of cross-attention mechanisms for climate features and soil features can offer a more comprehensive perspective of the combined influence of climate factors and soil conditions on crop growth.

#### 2.2.3. Yield Prediction Module

**Figure 4:**
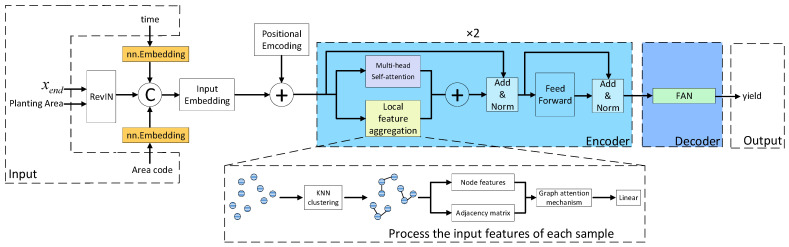
Yield prediction module.

This paper adopts the Transformer structure to design a yield prediction module, including the encoding of time and spatial information, adding position encoding, Encoder, and Decoder. Using the fused multi-source features, planting area, time, and study area code as inputs, the final output is the predicted yield. The module structure is shown in Figure 4.

The encoder has a total of 2 layers, each of which includes attention processing and feed forward neural network. The attention processing part uses the multi-head self-attention mechanism [34] and graph attention mechanisms to process features in parallel. The multi-head self-attention mechanism focuses on the global dependencies between features, while the graph attention mechanism enhances local features by aggregating feature information from neighboring nodes.

The decoder adopts a two-layer FAN [35] structure to learn the intricate nonlinear connection between the features processed by the encoder and the yield values to be predicted. The main calculation formula for FAN is shown in Equation (6).

(6) $$\emptyset \left(x\right)=[cos(W_{p}\cdot x)||sin(W_{p}\cdot x)||\sigma (B_{\bar{p}}+W_{\bar{p}}\cdot x)]$$

Among them, x is the input, ∅(x) is the output, $W_{p}$ and $W_{\bar{p}}$ are learnable projection matrices, $B_{\bar{p}}$ is a bias term, and σ represents the activation function. Compared with multi-layer perceptrons, introducing the Fourier series can ensure modeling capability while focusing on periodic patterns.

### 2.3. The Framework Prediction Process in This Paper

**Figure 5:**
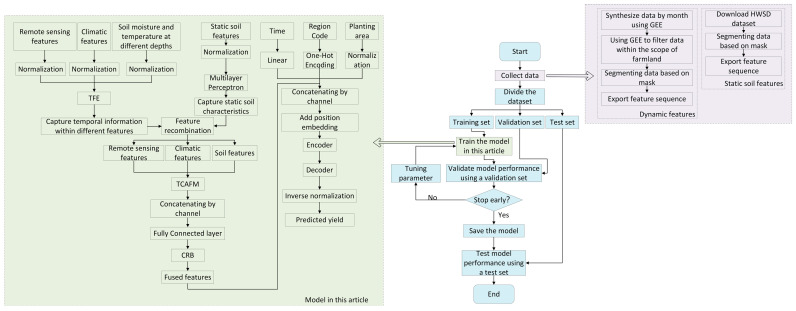
The flowchart corresponding to the framework of this paper.

Table 2 illustrates the prediction process of the framework in this paper, while Figure 5 shows the corresponding flowchart.

## 3. Results

For the methods presented in this paper and all the comparison methods involved, this paper used data from 196 research areas in 2021 and 2022 as the test set, and divided the remaining data from 2001 to 2020 into training and validation sets in an 8:2 ratio. We ensured consistent data partitioning results by setting random seeds. The model in this paper was trained using the Adam optimizer, with a batch size of eight, and a learning rate of 1 × 10^−3^, and 1000 epochs. An early stopping strategy and automatic adjustment of learning rate were employed. Training will be halted if there is no improvement in the loss on the validation set for 20 consecutive epochs, triggering an early stop. This early stopping strategy can effectively prevent model over fitting and ensure the model with optimal performance on the validation set is preserved. We used the ReduceLROnPlateau scheduler to automatically adjust the learning rate, reducing it to 90% when the validation loss does not improve within 10 epochs.

This paper selected four indicators to evaluate the effectiveness of the framework: Mean Absolute Error (MAE), Root Mean Square Error (RMSE), Mean Absolute Percentage Error (MAPE) and R-squared (R^2^).

### 3.1. Performance Comparison

**Figure 6:**
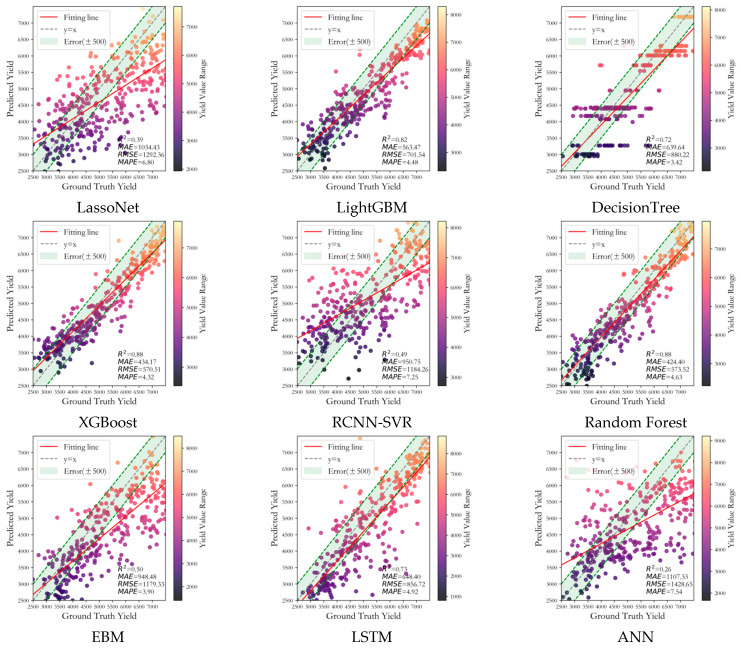

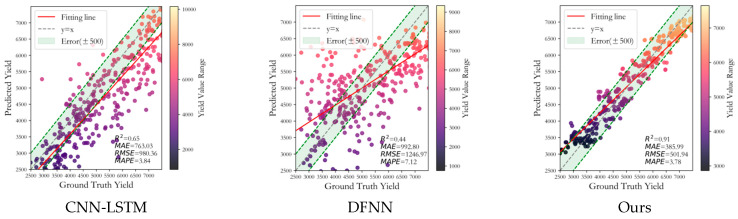
Display of prediction results using different methods.

**Figure 7:**
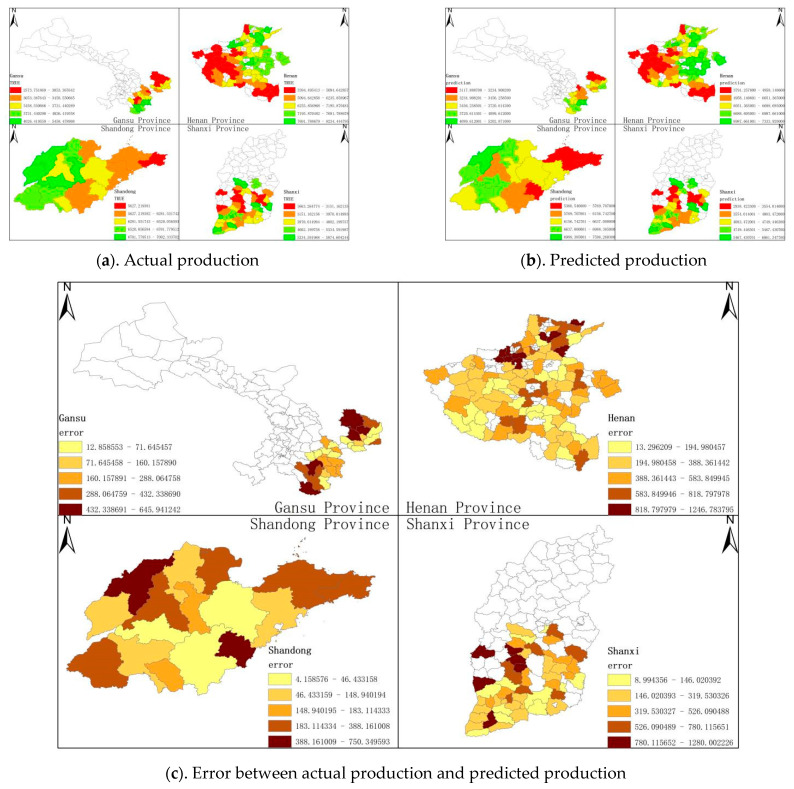
Display of 2021 winter wheat yield prediction results for various research regions based on the model presented in this paper.

To evaluate the effectiveness of the winter wheat yield forecasting framework with triple cross-attention for multi-source data fusion proposed in this paper, it has been compared with 11 other methods, including LassoNet [36], XGBoost [37], LSTM [37], ANN [37], Random Forest [37], Decision Tree [38], RCNN-SVR [39], EBM [40], CNN-LSTM [41], DFNN [42] and LightGBM [43]. Table 3 and Figure 6 display the results of the comparison. Figure 7 also shows the actual winter wheat yield in each study area in 2021, the predicted yield obtained by our method, and the error of both.

### 3.2. The Importance of Various Growth Phases of Winter Wheat in Yield Forecasting

**Figure 8:**
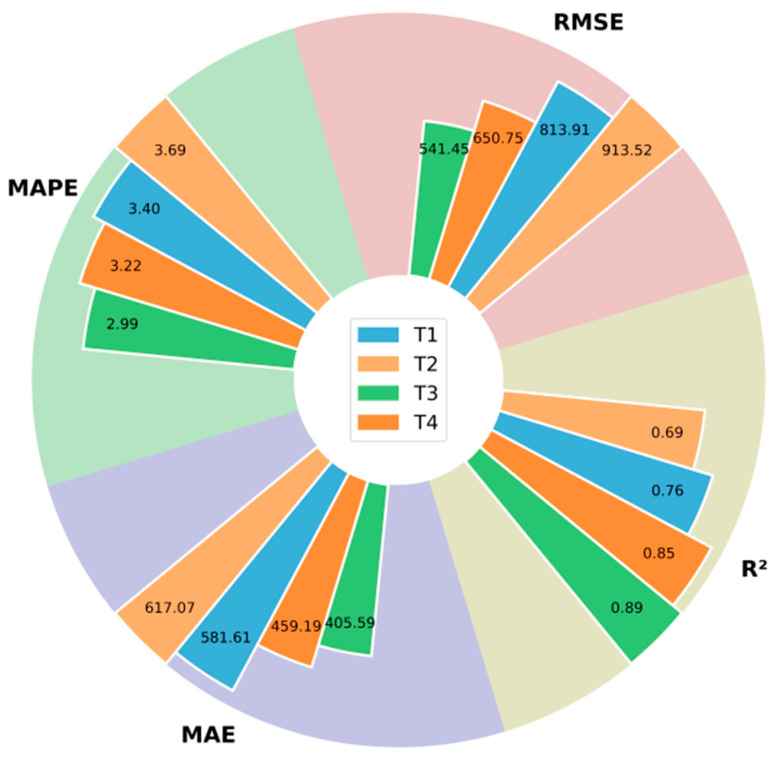
Display of yield prediction results using features of different growth stages.

To analyze the influence of different stage characteristics on yield prediction results during the winter wheat development period, this paper divides the growing season into four stages: emergence-tillering, winter dormancy stage, jointing-heading, and heading-maturity. Table 4 presents the detailed information for each stage [44]. Table 5 and Figure 8 show the results of using the characteristics of different growth stages of winter wheat for yield prediction.

### 3.3. The Effect of Time Window Length on Yield Prediction

**Figure 9:**
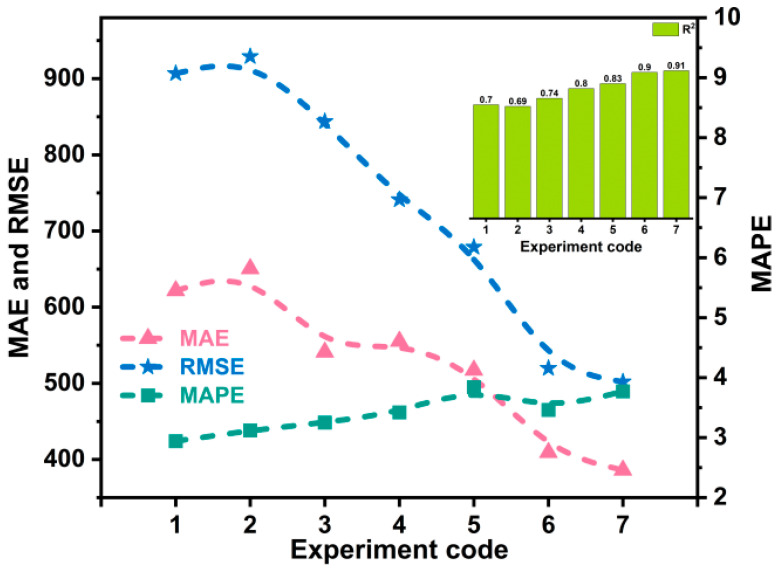
Display of prediction results using time windows of different lengths.

To further evaluate the model’s predictive performance, this paper used the features from the first 3, 4, 5, 6, 7, and 8 months of the growth period as inputs. Table 6 and Figure 9 display the predicted results.

### 3.4. Ablation Experiment

To fully demonstrate the model’s improvement, this paper collected data from a coarse-grained perspective and verified it with examples from the Shandong and Gansu provinces. We used the data from 46 research areas in these two provinces from 2021 to 2022 as the test set, and divided the remaining data from 2001 to 2020 into training and validation sets in an 8:2 ratio. We extracted winter wheat planting areas within the province using the annual plant functional type classification scheme (Land cover type 5) in MODIS land cover type product (MCD12Q1), and used it as a mask to obtain the satellite remote sensing data, climate data, and soil data of winter wheat planting areas across various cities, districts (counties). We added the Mean Squared Prediction Error (MSPE) to provide additional evidence of the improvement of the model.

#### 3.4.1. The Ablation Experiment of TFE and Graph Attention Mechanism

**Figure 10:**
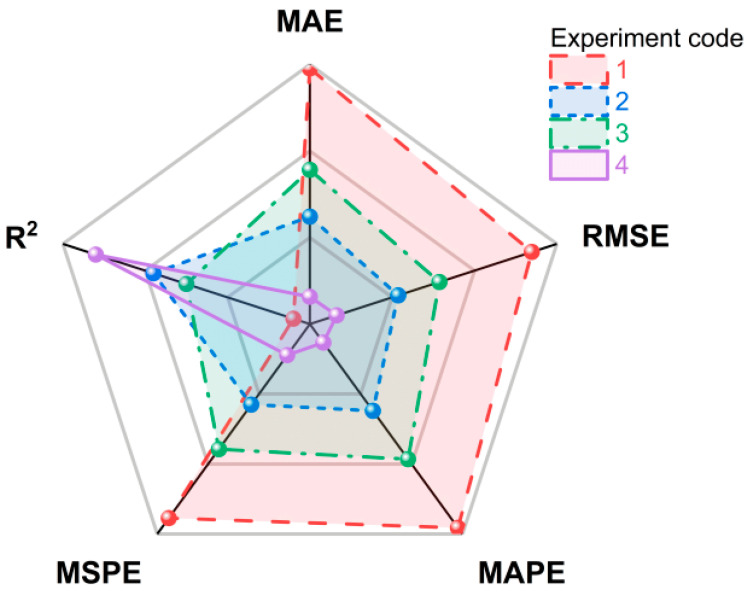
Display of ablation experiment results on TFE and graph attention mechanism.

To examine the effectiveness of the TFE and the applied graph attention mechanism, the following ablation experiments were designed. Experiment 1 represents the absence of the TFE and graph attention mechanism, Experiment 2 represents the presence of the TFE but without the graph attention mechanism, Experiment 3 represents the absence of the TFE but with the graph attention mechanism, and Experiment 4 is the model proposed in this paper. Table 7 and Figure 10 display the experimental results.

#### 3.4.2. The Ablation Experiment of TCAFM

**Figure 11:**
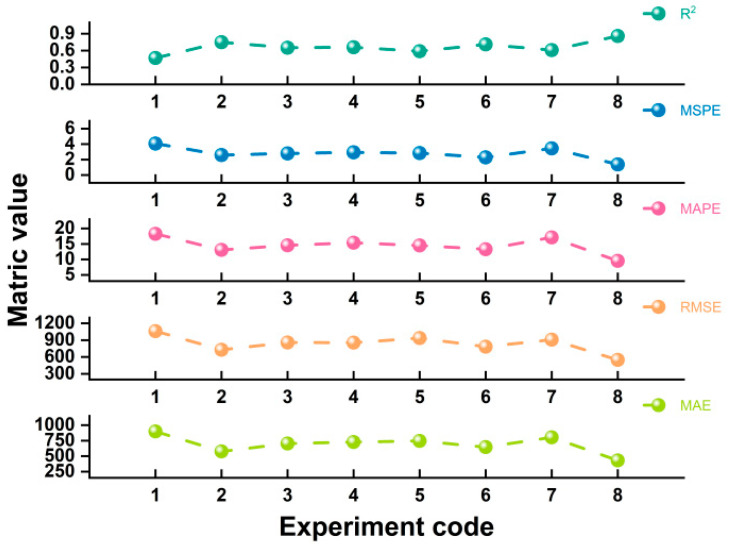
Display of ablation experiment results of TCAFM.

To confirm the effectiveness of the TCAFM, the following ablation experiments were developed to validate the importance of multi-source data fusion for yield forecasting tasks while verifying the effectiveness of the TCAFM. Among them, Experiment 1, Experiment 2, and Experiment 3 represent using only satellite remote sensing features, soil features, and climate features as model inputs, respectively. Experiment 4, Experiment 5, Experiment 6, and Experiment 7 represent using four methods of concatenation, addition, average, and maximum to fuse three types of features as model inputs, respectively. Experiment 8 is the method proposed in this paper. Table 8 and Figure 11 display the experimental results.

### 3.5. Efficiency of the Model

This paper compares the efficiency of different methods using inference time, model storage size, and parameters. The experimental results are shown in Table 9.

## 4. Discussion

### 4.1. Analysis of Comparative Test Results

Table 3 and Figure 6 show the evaluation metrics corresponding to the prediction results obtained by our model and 11 comparative methods, including XGBoost, EBM, etc. From the results, it is clear that the prediction outcomes generated by the model in this paper are the most reliable, with MAE, RMSE, and R^2^ of 385.99 kg/hm^2^, 501.94 kg/hm^2^, and 0.91, respectively. Next are ensemble approaches based on the decision tree such as XGBoost (MAE = 434.16 kg/hm^2^, RMSE = 570.51 kg/hm^2^, R^2^ = 0.88) and Random Forest (MAE = 424.4 kg/hm^2^, RMSE = 573.52 kg/hm^2^, R^2^ = 0.88), which indicate that our model has a strong yield prediction capability and can adaptively capture important information from multi-source features to achieve a yield prediction.

### 4.2. Analysis of the Importance of Different Winter Wheat Growth Phases in Predicting Yield

Table 5 and Figure 8 present the results produced by the model in this study using features from different growth phases of winter wheat for forecasting yield. The results indicate that the most accurate yield prediction can be obtained by using the features corresponding to the jointing-heading, followed by the characteristics from the heading-maturity. The MAE of the predicted results obtained using the features corresponding to the jointing-heading were 176.02 kg/hm^2^, 211.48 kg/hm^2^, and 53.6 kg/hm^2^ lower than those obtained using the emergence-tillering, winter dormancy stage, and heading-maturity, respectively. This may be because after the previous tillering, the number of spikes that wheat will form in the future has been basically determined. The more spikes, the more grains will be obtained. The number of spikes directly affects the final yield. Secondly, this stage provides the material foundation for the subsequent milk stage. The better the crop’s condition and the more nutrients accumulated through photosynthesis during this stage, the fuller the grains will be in the later stage. The level of grain plumpness is strongly correlated with the final yield.

### 4.3. Analysis of the Effect of Time Window Length on Yield Prediction

Table 6 and Figure 9 show the evaluation metrics corresponding to the prediction results of our model using various length time windows. The experimental results show that as the time window length increases further, the predicted yield by our method approaches the true yield constantly and error indicators such as MAE and RMSE decrease continuously. Using the approach outlined in this study, the MAE and RMSE of the predicted results obtained from the features of the first 8 months of the winter wheat growth period differ by 23.2 kg/hm^2^ and 17.72 kg/hm^2^, respectively, compared to the predicted results obtained from the complete growth period. This indicates that our model can predict the final yield of winter wheat nearly a month before harvest.

### 4.4. Analysis of Ablation Experimental Results of TFE and Graph Attention Mechanism

Table 7 and Figure 10 show the performance of our model with and without the TFE and graph attention mechanism. The experimental results indicate that by adding the TFE and graph attention mechanism, the predictive ability of our model can be significantly improved. Compared with the model without the TFE and graph attention mechanism (MAE = 695.39 kg/hm^2^, RMSE = 904.36 kg/hm^2^), the model with the TFE alone (MAE = 524.12 kg/hm^2^, RMSE = 660.92 kg/hm^2^), the model with the graph attention mechanism alone (MAE = 578.24 kg/hm^2^, RMSE = 736.13 kg/hm^2^), and the model with both (MAE = 431.47 kg/hm^2^, RMSE = 548.98 kg/hm^2^), the MAE decreases by about 24.6%, 16.8%, and 37.9%, and the RMSE decreases by about 26.9%, 18.6%, and 39.3%, respectively. This is due to the effective extraction of temporal features from different input features by the TFE, as well as the effectiveness of the graph attention mechanism in enhancing local information in the fused features.

### 4.5. Analysis of Ablation Experiment Results of TCAFM

Table 8 and Figure 11 show the prediction results obtained by our model using single source features and multi-source features fused by different fusion methods. The experimental results indicate that using a single source feature is not sufficient to achieve more accurate yield prediction. Because multiple factors influence crop yield, considering the impact of various factors is crucial when completing yield forecasting tasks. The experimental results also demonstrated that using the TCAFM (MAE = 431.47 kg/hm^2^, RMSE = 548.98 kg/hm^2^) can achieve better prediction results compared to four fusion strategies: channel splicing fusion (MAE = 728.66 kg/hm^2^, RMSE = 855.94 kg/hm^2^), additive fusion (MAE = 744.87 kg/hm^2^, RMSE = 937.06 kg/hm^2^), weighted average (MAE = 645.88 kg/hm^2^, RMSE = 782.22 kg/hm^2^), and maximum fusion (MAE = 803.01 kg/hm^2^, RMSE = 908.57 kg/hm^2^). The R2 obtained using the TCAFM proposed in this paper is 0.2, 0.27, 0.15, and 0.25 higher than those obtained using the four fusion strategies mentioned above. The higher the R2, the more accurate the model’s prediction. This is due to the fact that the fusion strategy in this paper takes into account the comprehensive relationships among features from various sources, thereby achieving the maximum utilization of features.

### 4.6. Analysis of Model Efficiency Experiment Results

From Table 9, it can be seen that in terms of inference time, it may be due to the complex model structure that the inference time of our method is longer, much higher than other methods. In terms of model storage size, the storage space required by our method is at a relatively low level among all methods, indicating that our model has certain storage advantages and practical deployment capabilities. In terms of the parameters, the method proposed in this paper has a large number of parameters, indicating its strong learning ability.

### 4.7. Discussion Summary

This paper uses a combination of coarse-grained and fine-grained datasets to validate the effectiveness of the model. The comparative experiment uses fine-grained data, while the ablation experiment uses coarse-grained data. Through this combination, it can be fully demonstrated that the model in this paper has a strong predictive ability.

To begin with, to assess the performance of the yield prediction framework presented in this study, it was contrasted with 11 different methods. The MAE, RMSE, and other indicators of the model in this paper outperformed those of the alternative methods, demonstrating the model’s effectiveness.

Secondly, to investigate how various development phases of winter wheat affect yield, this paper divides the complete growth period into four stages and uses the features of each stage as input of the framework of this paper. The predicted results are used to measure the extent to which the characteristics of different stages impact yield. Analyzing the experimental results reveals that the features of the jointing-heading stage have the greatest influence on the final yield.

Thirdly, to understand the predictive ability of the framework in this paper, the features of different time window lengths were used as model inputs. According to the findings, the model presented in this paper can provide relatively accurate predictions one month prior to the wheat harvest.

Furthermore, the ablation experiment on TFE and graph attention mechanism shows that the TFE can effectively capture the temporal relationships in the multi-source features of the input, while the graph attention mechanism can enhance the local information in the fused features, thereby improving the utilization of input features.

Moreover, the ablation experiment on the TCAFM demonstrates that relying on a single source feature does not yield reliable prediction results. It also shows that the TCAFM provides more reliable predictions compared to other fusion methods, thus proving its effectiveness.

Finally, our model has superior performance, but requires longer inference time and a larger number of parameters. By combining its predictive performance, it can be seen that it has a strong modeling ability and can capture complex relationships in features. However, in application scenarios that require high real-time performance, further optimization of inference efficiency is needed. In the future, the following strategies can be explored for optimization. Firstly, replace the backbone network with a lightweight architecture and use efficient operations such as depthwise separable convolution to reduce computational complexity and storage requirements while minimizing the impact on model performance. Secondly, we will further explore how to strike a balance between accuracy, latency, and energy consumption to adapt to actual deployment on edge devices.

## 5. Conclusions

To address the issues in current yield prediction methods, such as the heavy reliance on domain expertise, poor model generalization, and the manual feature selection process that consumes a large amount of computational resources, this paper proposes a winter wheat yield prediction framework with triple cross-attention and multi-source data fusion. This framework consists of three modules, namely a multi-source data processing module, a multi-source feature fusion module, and a yield prediction module. We used the framework proposed in this paper to predict the winter wheat yield per unit area from 2021 to 2022 at the municipal level in Shandong Province, as well as at some county levels in Shanxi Province, Henan Province, and Gansu Province. The results indicate that compared with other methods, our method can achieve better prediction results. Meanwhile, the proposed framework’s effectiveness was further demonstrated through ablation experiments using coarse-grained data. However, the framework of this paper still has problems such as a long running time, a large number of parameters, and poor interpretability. Therefore, in future work, we will further simplify the model, improve its interpretability, lightweight the model as much as possible without reducing its performance, and clarify the extent to which different variables contribute to the prediction results, pursuing a balance between high interpretability and strong predictive ability.

## Figures and Tables

**Table 1:** The description of multi-source data.

Data	Detailed Variables	Temporal Resolution	Data Sources
Ground statistical data [28]	Yield per unit area, Sowing area	year	Statistical yearbook
Satellite remote sensing data [29]	NDVI, EVI	month	MOD13Q1
Climate data [30]	Evapotranspiration, Net long-wave radiation flux, Net short-wave radiation flux, Surface pressure, Total precipitation rate, Near-surface air temperature, Near-surface wind speed	month	FLDAS
Soil data [30,31]	Moisture and temperature of soil at different depths	month	FLDAS
	Reference bulk density, organic carbon, pH value, sand fraction, clay fraction, silt fraction, cation exchange capacity of the entire soil surface, and cation exchange capacity of the clay portion for both surface and deep soil	constant	National Cryosphere Desert Data Center
Spatio-Temporal data [28]	Time (year)	year	2001–2022
	Space (region code)	constant	196 regions in total

**Table 2:** Pseudo code.

A Winter Wheat Yield Prediction Framework with Triple Cross-Attention and Multi-Source Data Fusion	
Input: Multi-source features during the winter wheat growing season $\;X^{multi}=\{x^{rs},x^{cl},x^{so-d},x^{so-s}\}$, Time (T), Region Code (R), Planting Area (PA)	
Output: Predicting yield $\hat{y}$ and evaluation indicators	
Step 1	Construct dataset $D={\left\{\left(X_{i},y_{i}\right)\right\}}_{i=1}^{N}$ from multi-source data
Step 2	$D\to D_{train},D_{val},D_{test}$
Step 3	For each epoch t=1,2,...,1000:
(a)	$X_{i}\leftarrow RevIN(X_{i})$
(b)	$F^{rs}\leftarrow TFE(x^{rs}),F^{cl}\leftarrow TFE(x^{cl}),F^{so-d}\leftarrow TFE(x^{so-d}),F^{so-s}\leftarrow CRB(x^{so-s}),F^{so}\leftarrow concat(F^{so-d},F^{so-s})$
(c)	$F^{fused}\leftarrow TCAFM(F^{rs},F^{cl},F^{so})$
(d)	$F^{time}\leftarrow Linear(T),\;F^{region}\leftarrow OneHot(R),F^{area}\leftarrow Normalize(PA)$
(e)	$X\leftarrow Concat(F^{time},F^{region},F^{area},F^{fused})$
(f)	$X^{pos}\leftarrow X+Position\;Encoding$
(g)	${\hat{y}}_{j}\leftarrow Decoder(Encoder(X^{pos}))$
(h)	$L_{val}=\frac{1}{M}\sum_{j=1}^{M}\left|{\hat{y}}_{j}-y_{j}\right|$
(i)	If $L_{val}$ does not decrease for 40 epoches:
(j)	Save model and Early stop
(k)	Test the saved model
(l)	Compute and output metrics (MAE, RMSE, MAPE, MSPE, and $R^{2}$)

**Table 3:** Comparison of evaluation indicators for predicting results using different methods.

Methods	MAE (kg/hm^2^)	RMSE (kg/hm^2^)	MAPE (%)	R^2^
LassoNet [36]	1034.43	1292.36	6.8	0.39
LightGBM [43]	563.47	701.54	4.48	0.82
DecisionTree [38]	639.64	880.22	**3.42**	0.72
XGBoost [37]	434.17	570.51	4.32	0.88
RCNN-SVR [39]	950.75	1184.26	7.25	0.49
Random Forest [37]	424.4	573.52	4.63	0.88
EBM [40]	948.48	1179.33	3.9	0.5
LSTM [37]	648.4	856.72	4.92	0.73
ANN [37]	1107.33	1428.65	7.54	0.26
CNN-LSTM [41]	763.03	980.36	3.84	0.65
DFNN [42]	992.8	1246.97	7.12	0.44
Ours	**385.99**	**501.94**	3.78	**0.91**

Note: The bold indicates the optimal result.

**Table 4:** Information on different stages of growth.

Stage Abbreviation	T1	T2	T3	T4
Growth period	emergence-tillering	winter dormancy stage	jointing-heading	heading-maturity
Corresponding month	October to November	December to February of the following year	March to April of the following year	May to June of the following year

**Table 5:** Yield prediction results using features of different growth stages.

	Indicators	MAE (kg/hm^2^)	RMSE (kg/hm^2^)	MAPE (%)	R^2^
Stage					
T1		581.61	813.91	3.4	0.76
T2		617.07	913.52	3.69	0.69
T3		405.59	541.45	2.99	0.89
T4		459.19	650.75	3.22	0.85

**Table 6:** Prediction results using time windows of different lengths.

Number	Time Window									MAE (kg/hm^2^)	RMSE (kg/hm^2^)	MAPE (%)	R^2^
	Oct.	Nov.	Dec.	Jan.	Feb.	Mar.	Apr.	May	Jun.				
1	√	√	√							621.69	906.88	2.94	0.7
2	√	√	√	√						650.42	929.13	3.12	0.69
3	√	√	√	√	√					541.01	843.74	3.25	0.74
4	√	√	√	√	√	√				555.19	741.11	3.42	0.8
5	√	√	√	√	√	√	√			517.21	678.84	3.84	0.83
6	√	√	√	√	√	√	√	√		409.19	519.66	3.46	0.9
7	√	√	√	√	√	√	√	√	√	385.99	501.94	3.78	0.91

Note: The “√” symbol indicates the use of data for that month.

**Table 7:** Experimental results of TFE and graph attention mechanism ablation.

Number	TFE	Graph Attention Mechanism	MAE (kg/hm^2^)	RMSE (kg/hm^2^)	MAPE (%) × 100	MSPE (%^2^) × 100	R^2^
1	-	-	695.39	904.36	14.82	3.31	0.62
2	√	-	524.12	660.92	11.48	1.96	0.79
3	-	√	578.24	736.13	12.86	2.49	0.75
4	Ours		431.47	548.98	9.53	1.37	0.86

Note: The “-” symbol indicates that no module is used. The “√” symbol indicates that the use of this module.

**Table 8:** The ablation experiment results of TCAFM.

Number	Remote Sensing	Soil	Climate	Fusion Method	MAE (kg/hm^2^)	RMSE (kg/hm^2^)	MAPE (%) × 100	MSPE (%^2^) × 100	R^2^
1	√	-	-	-	899.23	1058.05	18.25	4.06	0.47
2	-	√	-	-	576.81	728.28	13.08	2.59	0.75
3	-	-	√	-	704.33	857.3	14.54	2.79	0.65
4	√	√	√	concat	728.66	855.94	15.39	2.93	0.66
5	√	√	√	add	744.87	937.06	14.51	2.84	0.59
6	√	√	√	avg	645.88	782.22	13.29	2.29	0.71
7	√	√	√	max	803.01	908.57	17.11	3.45	0.61
8	Ours				431.47	548.98	9.53	1.37	0.86

Note: The “-” symbol indicates that no fusion method has been used for feature fusion. The “√” symbol indicates that the use of the data.

**Table 9:** Experimental results of efficiency analysis of different models.

	Inference Time (s)	Model Storage Size (MB)	Parameters (M)
LassoNet [36]	0.043058	8.4622	0.039
LightGBM [43]	0.076627	1.5070	0.012
DecisionTree [38]	0.001994	0.0084	0.001
XGBoost [37]	0.025452	1.9506	0.053
RCNN-SVR [39]	0.095112	26.0975	0.003
Random Forest [37]	0.033336	31.5588	0.459
EBM [40]	0.051753	23.0054	0.124
LSTM [37]	0.051126	1.36	0.116
ANN [37]	0.165146	0.94	0.079
CNN-LSTM [41]	0.070710	0.3	0.023
DFNN [42]	0.001002	0.1302	0.031
Ours	0.244363	1.4733	0.356

## Data Availability

The datasets generated during and/or analyzed during the current study are available from the corresponding author on reasonable request.

## References

[1] Zhao Y., Xiao D., Bai H. (2024). The simultaneous prediction of yield and maturity date for wheat–maize by combining satellite images with crop model. J. Sci. Food Agric..

[2] Zhao Y., Xiao D., Bai H., Tang J., Liu D.L., Qi Y., Shen Y. (2022). The prediction of wheat yield in the North China Plain by coupling crop model with machine learning algorithms. Agriculture.

[3] Akumaga U., Gao F., Anderson M., Dulaney W.P., Houborg R., Russ A., Hively W.D. (2023). Integration of remote sensing and field observations in evaluating DSSAT model for estimating maize and soybean growth and yield in Maryland, USA. Agronomy.

[4] Yang M., Wang G., Wu S., Block P., Lazin R., Alexander S., Lala J., Haider M.R., Dokou Z., Atsbeha E.A. (2023). Seasonal prediction of crop yields in Ethiopia using an analog approach. Agric. For. Meteorol..

[5] Yang M., Wang G. (2023). Heat stress to jeopardize crop production in the US Corn Belt based on downscaled CMIP5 projections. Agric. Syst..

[6] Kaur H., Huggins D.R., Carlson B., Stockle C., Nelson R. (2022). Dryland fallow vs flex-cropping decisions in inland Pacific Northwest of USA. Agric. Syst..

[7] Bregaglio S., Ginaldi F., Raparelli E., Fila G., Bajocco S. (2023). Improving crop yield prediction accuracy by embedding phenological heterogeneity into model parameter sets. Agric. Syst..

[8] Fei S., Xiao S., Zhu J., Xiao Y., Ma Y. (2024). Dual sampling linear regression ensemble to predict wheat yield across growing seasons with hyperspectral sensing. Comput. Electron. Agric..

[9] Li Q.C., Xu S.W., Zhuang J.Y., Liu J.J., Zhuo Y., Zhang Z.X. (2023). Ensemble learning prediction of soybean yields in China based on meteorological data. J. Integr. Agric..

[10] Sun X., Zhang P., Wang Z. (2024). Potential of multi-seasonal vegetation indices to predict rice yield from UAV multispectral observations. Precis. Agric..

[11] Li Y., Zeng H., Zhang M., Wu B., Zhao Y., Yao X., Cheng T., Qin X., Wu F. (2023). A county-level soybean yield prediction framework coupled with XGBoost and multidimensional feature engineering. Int. J. Appl. Earth Obs..

[12] Yu W., Yang G., Li D., Zheng H., Yao X., Zhu Y., Cao W., Qiu L., Cheng T. (2023). Improved prediction of rice yield at field and county levels by synergistic use of SAR, optical and meteorological data. Agr. For. Meteorol..

[13] de Sá Leitão D.A.H., Sharma A.K., Singh A., Sharma L.K. (2023). Yield and plant height predictions of irrigated maize through unmanned aerial vehicle in North Florida. Comput. Electron. Agric..

[14] Zhang H., Zhang Y., Liu K., Lan S., Gao T., Li M. (2023). Winter wheat yield prediction using integrated Landsat 8 and Sentinel-2 vegetation index time-series data and machine learning algorithms. Comput. Electron. Agric..

[15] Wang Y.Q., Leng P., Shang G.F., Zhang X., Li Z.L. (2023). Sun-induced chlorophyll fluorescence is superior to satellite vegetation indices for predicting summer maize yield under drought conditions. Comput. Electron. Agric..

[16] Skobalski J., Sagan V., Alifu H., Al Akkad O., Lopes F.A., Grignola F. (2024). Bridging the gap between crop breeding and GeoAI: Soybean yield prediction from multispectral UAV images with transfer learning. ISPRS J. Photogramm. Remote Sens..

[17] Cheng E.H., Zhang B., Peng D.L., Zhong L.H., Yu L., Liu Y., Xiao C.C., Li C.J., Li X.Y., Chen Y. (2022). Wheat yield estimation using remote sensing data based on machine learning approaches. Front. Plant Sci..

[18] Wang J., Wang P., Tian H., Tansey K., Liu J., Quan W. (2023). A deep learning framework combining CNN and GRU for improving wheat yield estimates using time series remotely sensed multi-variables. Comput. Electron. Agric..

[19] Guo Y., Xiao Y., Hao F., Zhang X., Chen J., Beurs K., He Y., Fu Y.H. (2023). Comparison of different machine learning algorithms for predicting maize grain yield using UAV-based hyperspectral images. Int. J. Appl. Earth Obs..

[20] Feng A., Zhou J., Vories E., Sudduth K.A. (2024). Prediction of cotton yield based on soil texture, weather conditions and UAV imagery using deep learning. Precis. Agric..

[21] Fathi M., Shah-Hosseini R., Moghimi A. (2023). 3D-ResNet-BiLSTM Model: A Deep Learning Model for County-Level Soybean Yield Prediction with Time-Series Sentinel-1, Sentinel-2 Imagery, and Daymet Data. Remote Sens..

[22] Tanabe R., Matsui T., Tanaka T.S.T. (2023). Winter wheat yield prediction using convolutional neural networks and UAV-based multispectral imagery. Field Crop. Res..

[23] Bi L., Wally O., Hu G., Tenuta A.U., Kandel Y.R., Mueller D.S. (2023). A transformer-based approach for early prediction of soybean yield using time-series images. Front. Plant Sci..

[24] Perich G., Turkoglu M.O., Graf L.V., Wegner J.D., Aasen H., Walter A., Liebisch F. (2023). Pixel-based yield mapping and prediction from Sentinel-2 using spectral indices and neural networks. Field Crop. Res..

[25] Cheng E., Wang F., Peng D., Zhang B., Zhao B., Zhang W., Hu J., Lou Z., Yang S., Zhang H. (2024). A GT-LSTM Spatio-Temporal Approach for Winter Wheat Yield Prediction: From the Field Scale to County Scale. IEEE Trans. Geosci. Remote Sens..

[26] Guo F., Wang P., Tansey K., Zhang Y., Li M., Liu J., Zhang S. (2024). A novel transformer-based neural network under model interpretability for improving wheat yield estimation using remotely sensed multi-variables. Comput. Electron. Agric..

[27] Kiran Kumar V., Ramesh K.V., Rakesh V. (2023). Optimizing LSTM and Bi-LSTM models for crop yield prediction and comparison of their performance with traditional machine learning techniques. Appl. Intell..

[28] National Bureau of Statistics of China (2024). China Statistical Yearbook. https://www.stats.gov.cn/sj/ndsj/.

[29] NASA Land Processes Distributed Active Archive Center (2021). MODIS/Terra Vegetation Indices 16-Day L3 Global 250 m SIN Grid V061. https://www.earthdata.nasa.gov/data/catalog/lpcloud-mod13q1-061.

[30] McNally A., Arsenault K., Kumar S., Shukla S., Peterson P., Wang S.G., Funk C., Peters-Lidard D.C., Verdin P.J. (2017). A land data assimilation system for sub-Saharan Africa food and water security applications. Sci. Data.

[31] Fischer G., Nachtergaele F., Prieler S., Teixeira E., Tóth G., Velthuizen H., Verelst L., Wiberg D. (2008). Global Agro-Ecological Zones Assessment for Agriculture (GAEZ 2008).

[32] Dong J., Fu Y., Wang J., Tian H., Fu S., Niu Z., Han W., Zheng Y., Huang J., Yuan W. (2020). Early season mapping of winter wheat in China based on Landsat and Sentinel images. Earth. Syst. Sci. Data.

[33] Shaker A., Maaz M., Rasheed H., Khan S., Yang M., Khan F.S. (2023). Swiftformer: Efficient additive attention for transformer-based real-time mobile vision applications. Proceedings of the IEEE/CVF International Conference on Computer Vision.

[34] Vaswani A., Shazeer N., Parmar N., Uszkoreit J., Jones L., Gomez A.N., Kaiser L., Polosukhin I. (2017). Attention is all you need. Adv. Neural Inf. Process. Syst..

[35] Dong Y., Li G., Tao Y., Jiang X., Zhang K., Li J., Deng J., Su J., Zhang J., Xu J. (2024). FAN: Fourier Analysis Networks. arXiv.

[36] Lemhadri I., Ruan F., Abraham L., Tibshirani R. (2021). Lassonet: A neural network with feature sparsity. J. Mach. Learn. Res..

[37] von Bloh M., Júnior R.S.N., Wangerpohl X., Saltık A.O., Haller V., Kaiser L., Asseng S. (2023). Machine learning for soybean yield forecasting in Brazil. Agric. For. Meteorol..

[38] Raj S., Patle S., Rajendran S. (2022). Predicting Crop Yield Using Decision Tree Regressor. Proceedings of the International Conference on Knowledge Engineering and Communication Systems (ICKES).

[39] Zhang Y., Li Q. (2020). A regressive convolution neural network and support vector regression model for electricity consumption forecasting. Advances in Information and Communication, Proceedings of the 2019 Future of Information and Communication Conference (FICC), San Francisco, CA, USA, 14–15 March 2019.

[40] Celik M.F., Isik M.S., Taskin G., Erten E., Camps-Valls G. (2023). Explainable artificial intelligence for cotton yield prediction with multisource data. IEEE Geosci. Remote Sens. Lett..

[41] Wang X., Yang Y., Zhao X., Huang M., Zhu Q. (2023). Integrating field images and microclimate data to realize multi-day ahead forecasting of maize crop coverage using CNN-LSTM. Int. J. Agric. Biol. Eng..

[42] Khaki S., Wang L. (2019). Crop yield prediction using deep neural networks. Front. Plant Sci..

[43] Pujitha B., Radha K., Samitha K., Prathyusha N., Vasanthi P. (2024). Crop Prediction from Soil Parameters using Light Ensemble Learning Model. Proceedings of the 2024 11th International Conference on Signal Processing and Integrated Networks (SPIN).

[44] Li L.C., Wang B., Feng P.Y., Liu D.L., He Q.S., Zhang Y.J., Wang Y.K., Li S.Y., Lu X.L., Yue C. (2022). Developing machine learning models with multi-source environmental data to predict wheat yield in China. Comput. Electron. Agric..

